# A patient with an ectopic sphenoid bone TSH secretory adenoma: Case report and review of the literature

**DOI:** 10.3389/fendo.2022.961256

**Published:** 2022-08-08

**Authors:** Shejil Kumar, Cun An Phang, Huajing Ni, Terrence Diamond

**Affiliations:** Endocrinology Department, St George Public Hospital, Sydney, NSW, Australia

**Keywords:** thyrotoxicosis, thyroid-stimulating hormone, TSHoma, ectopic TSHoma, thyrotropinoma, ectopic thyrotropin (TSH) secreting pituitary adenoma

## Abstract

Ectopic thyroid-stimulating hormone (TSH)oma located outside the sella turcica is exceedingly rare and can be associated with significant diagnostic delay. The clinical presentation depends on the anatomical location and size of the ectopic tumor and the degree of thyrotoxicosis. A 71-year-old woman presented with goiter and thyrotoxicosis. Initial investigations revealed elevated free thyroxine (fT4) and tri-iodothyronine (fT3) with inappropriately high-normal TSH. Assay interference was unlikely, pituitary magnetic resonance imaging (MRI) scan was reported as “normal,” and germline sequencing was negative for thyroid hormone receptor ß pathogenic variants. One year later, total thyroidectomy for enlarging symptomatic goiter and suspicious nodule revealed multifocal microscopic papillary thyroid carcinoma. Six years later, she presented to an ear, nose, and throat surgeon with nasal congestion, and a sphenoid bone mass was discovered on nasoendoscopy and imaging. Ectopic TSHoma was confirmed on surgical resection, and a review of the initial pituitary MRI scan revealed the mass which had initially been missed. This is the first reported case of an ectopic TSHoma located in the sphenoid bone. Ectopic TSHoma should be considered in patients with inappropriate TSH secretion when more common differentials are excluded including thyroid hormone resistance or pituitary TSHoma.

## Introduction

Pituitary adenomas originate from the adenohypophysis, and ectopic pituitary adenomas (EPAs) are extrasellar and are separate from the hypophysis and infundibulum (1, 2). EPA pathogenesis largely relates to the embryological development and migration of the adenohypophysis (1, 2). EPAs are rare with approximately 180 cases described according to a recent extensive literature review (1), and clinical manifestations vary widely, depending on their secretory profile, size, and anatomical location. Symptoms often mimic those of the skull base or nasopharyngeal tumors and occur due to mass effect on adjacent structures, resulting in visual disturbance, facial paraesthesia, headache, and nasal congestion (2, 3). Specifically, nasopharyngeal and sphenoid sinus EPAs commonly present with epistaxis and nasal congestion, while sphenoid bone EPAs can also present with headache and cranial neuropathy (4). According to a review of published cases, most EPAs (85%) are hormonally active, of which ACTH (36%), prolactin (28%), and GH (22%) secretions are the most common (3, 4). TSH-secreting EPAs (ectopic TSHomas) are the rarest, and to our knowledge, only 13 such cases have been reported in the literature to date (5–17). TSHomas manifest as secondary hyperthyroidism, with elevated serum free thyroxine (fT4) and tri-iodothyronine (fT3) and non-suppressed serum TSH, and can present clinically with symptoms and signs of thyrotoxicosis and diffuse goiter which can often lead to misdiagnosis or delayed diagnosis. We present the first report of an ectopic TSHoma located within the sphenoid bone in a 71-year-old woman. A review of all reported cases of ectopic TSHoma provides key insights into the presentation, diagnosis, and treatment of this rare and often missed disorder.

## Case description

A 71-year-old woman was referred to our endocrinology service in 2014 with a longstanding history of goiter for the past 10 years and recent intermittent dysphagia with solids without symptoms of thyrotoxicosis. She had a background of Hashimoto’s thyroiditis, was born in Australia, and had no prior neck irradiation or family history of thyroid-related conditions. She had no history of atrial fibrillation or osteoporosis. Examination confirmed a large, palpable non-obstructive goiter. She was normocardic and in normal sinus rhythm.

## Diagnosis, treatment, and outcomes

Thyroid function tests (TFTs) revealed elevated serum fT4 of 21.6 pmol/L (reference range: 9–19 pmol/L), high-normal fT3 of 5.8 pmol/L (reference range: 2.6–6.0 pmol/L), and inappropriately high-normal TSH of 3.79 mIU/L (reference range: 0.40–4.00 mIU/L). A review of previous biochemistry using different assays (including Roche and Abbott Architect) 6 years prior demonstrated a similar pattern of thyroid function derangement, and hence, assay interference was deemed unlikely. Other investigations in 2014 showed elevated anti-thyroid peroxidase antibodies of >1,000 IU/ml (reference range: 0–120 IU/ml) and elevated anti-thyroglobulin antibodies of 1,331 IU/ml (reference range: 0–80 IU/ml), consistent with Hashimoto’s thyroiditis. Serum TSH α-subunit level was considered normal at 1.04 IU/L (postmenopausal reference range: 0–1.3 IU/L). The remainder of the pituitary panel was unremarkable: postmenopausal range follicle-stimulating hormone (FSH) of 61.3 IU/L (reference range: 25–130 IU/L) and luteinizing hormone (LH) of 24.7 IU/L (reference range: 5–62 IU/L), insulin-like growth factor 1 (IGF-1) of 11 nmol/L (reference range: 10–28 nmol/L), prolactin of 302 mIU/L (reference range: 85–500 mIU/L), cortisol of 353 nmol/L (reference range: 138–650 nmol/L), and adrenocorticotrophin hormone (ACTH) of 7.1 pmol/L (reference range: 0–12 pmol/L). Further investigations were undertaken to elucidate the underlying cause for inappropriate TSH secretion. No pathogenic variants were detected on germline DNA polymerase chain reaction (PCR) and Sanger sequencing of exons 7–10 and flanking intronic sequences of the thyroid hormone receptor ß (*THRß*) gene. Magnetic resonance imaging (MRI) of the pituitary suggested a normal pituitary gland and unperturbed infundibulum, optic chiasm, and cavernous sinuses.

One year later, she underwent total thyroidectomy for progressively enlarging symptomatic goiter and suspicious 2.2 cm right inferior thyroid nodule of Bethesda category III on fine needle aspiration biopsy. Histopathology revealed four foci of microscopic papillary thyroid cancer (micro-PTC) ranging between 0.5 and 3 mm in diameter with clear resection margins and no evidence of lymphovascular invasion or extrathyroidal extension. Post-thyroidectomy radioactive iodine ablation was not required. Thyroxine replacement was commenced but subsequent TFTs showed persistently elevated or high-normal serum TSH despite increasing doses of thyroxine to as much as 1,200 mcg/week (2.0 mcg/kg/day).

**Figure 1:**
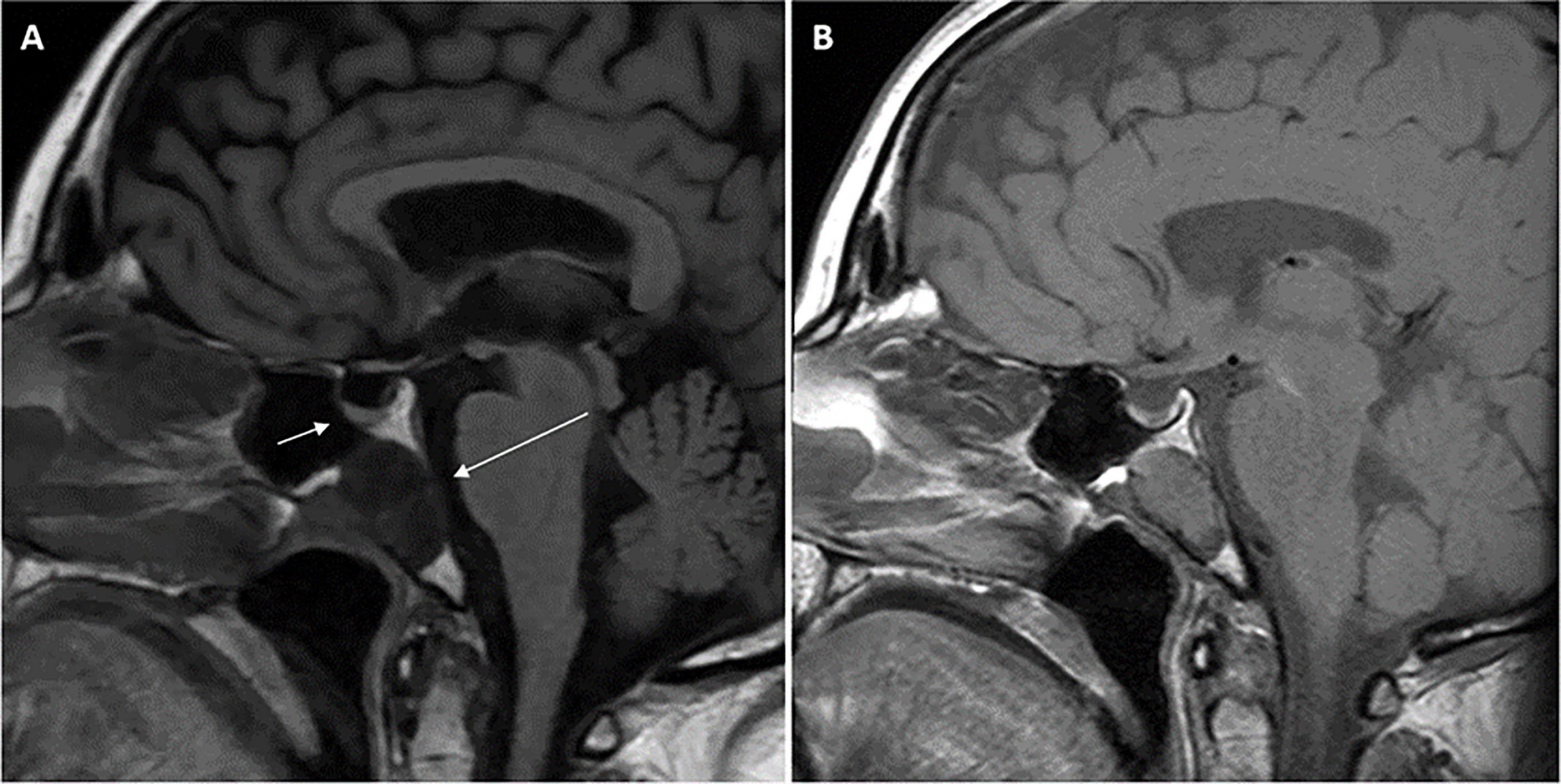
Magnetic resonance imaging (MRI) scans of the head demonstrating midline sphenoid bone mass. T1-weighted (FLAIR) sagittal view pituitary magnetic resonance imaging (MRI) scan performed in 2021 **(A)** demonstrating normal pituitary gland (short arrow) and adjacent midline T1-hypointense 3.0 cm × 2.3 cm × 2.3 cm mass (long arrow) located within the sphenoid bone extending to the clival, sphenoid sinus, and nasopharyngeal surfaces of the sphenoid bone with no extension into the sphenoid sinus or nasopharynx. Retrospective review of the T1-weighted sagittal view initial pituitary MRI scan performed in 2014 **(B)** demonstrated the same lesion within the sphenoid bone measuring 2.2 cm × 2.1 cm × 2.3 cm. Arrows are not included in **(B)** so the image is unperturbed and seen in the same way the radiologist viewed the scan.

**Figure 2:**
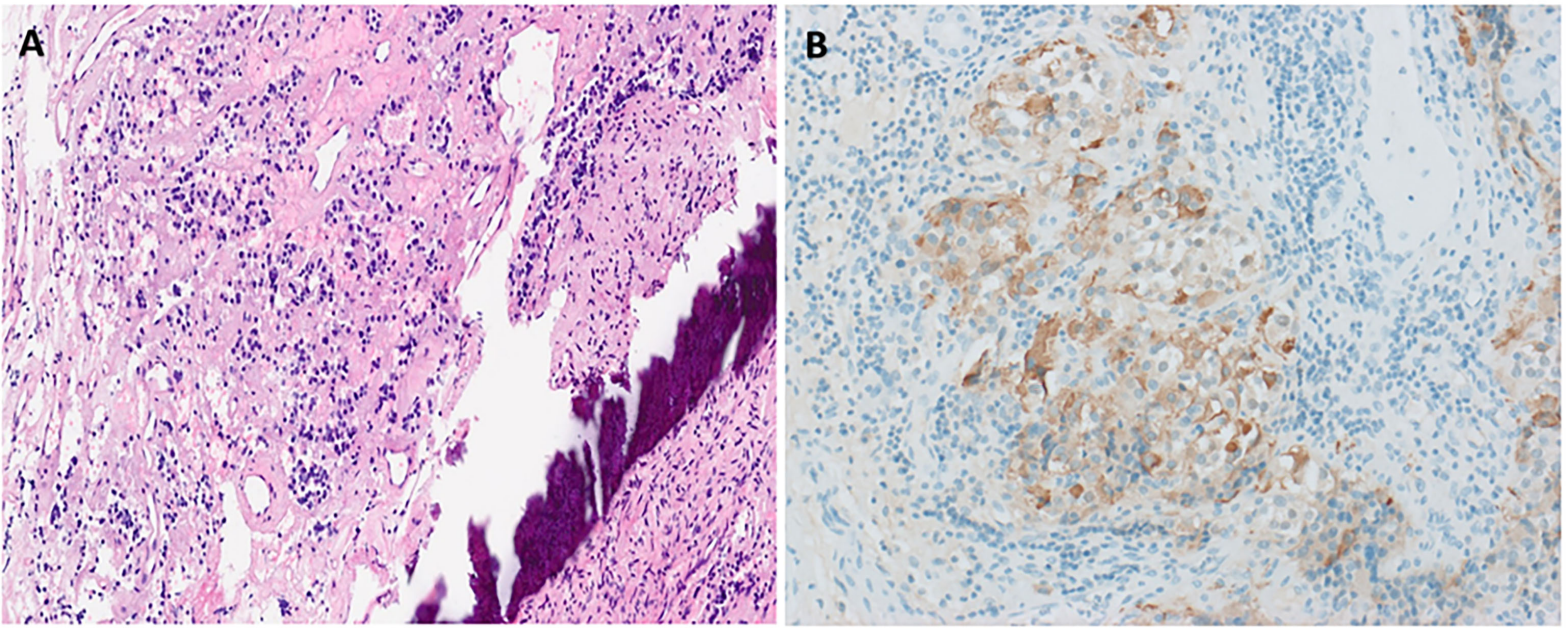
Histopathological confirmation of ectopic TSHoma. Histopathology (obtained from tumor resection) with hematoxylin and eosin staining **(A)** showing small irregularly shaped islands of cells separated by fibrovascular stroma beside the bone (dark purple streak). Cells have small amounts of lightly eosinophilic, granular cytoplasm and small–intermediate-sized round nuclei with no mitoses identified. Strong diffuse positive tumor cell staining on immunohistochemistry for TSH is also seen **(B)**.

She then presented 6 years later with nasal congestion to an ear, nose, and throat surgeon who suspected a nasopharyngeal mass on nasoendoscopy. Computed tomography (CT) of the paranasal sinuses showed a 2.6-cm × 1.4-cm × 2.5-cm lytic lesion expanding the sphenoid bone and separate from the sella turcica. A subsequent MRI scan of the head demonstrated a 3.0-cm × 2.3-cm × 2.3-cm well-marginated T2-hyperintense and T1-hypointense lesion with heterogeneous contrast enhancement located within the sphenoid bone without extension into the sphenoid sinus or nasopharynx (**Figure 1**). Endonasal endoscopic resection and tumor histopathology confirmed an ectopic pituitary TSH-producing neuroendocrine tumor with invasion of the clivus and nasopharyngeal mucosa (**Figure 2**). Tumor cells had small amounts of lightly eosinophilic, granular cytoplasm and small–intermediate-sized round nuclei without any mitoses identified and with a Ki67 index of 0.4%. Immunohistochemistry showed strong and diffuse staining for chromogranin A, synaptophysin, Pit1 transcription factor, TSH (**Figure 2**), and TSH α-subunit. Growth hormone and prolactin showed focal, weak staining, and transcription factors T-pit and SF1 were negative. Following tumor resection, her serum TSH decreased to 0.01 mIU/L. A retrospective review of her initial pituitary MRI scan images in 2014 revealed that a 2.2-cm × 2.1-cm × 2.3-cm mass within the sphenoid bone had initially been missed (**Figure 1**). At 5 months post-resection, thyroxine requirements were reduced to 800 mcg/week (1.3 mcg/kg/day) with normalized TFTs.

## Timeline

**Table:** 

February 2008	First biochemical evidence of TSH hypersecretion
March 2014	Endocrinology institution referral
October 2014	Initial pituitary MRI scan reported as “normal” Negative genetic testing for *THRß* mutation
June 2015	Total thyroidectomy for obstructive goiter and suspicious thyroid nodule
August 2021	Ear, nose, and throat surgeon referral for investigation of nasal congestion Nasoendoscopy suggested nasopharyngeal mass
September 2021	MRI head scan identified sphenoid bone mass
October 2021	Resection of sphenoid bone mass and confirmation of the diagnosis of ectopic TSHoma

## Discussion

The pathogenesis of EPAs relates to the embryological development of the hypophysis (13). Between the fourth and eighth week of gestation, Rathke’s pouch develops from the ascending invagination of extracranial ectoderm from the primitive oral cavity, and it fuses with the descending neuroectoderm to form the adenohypophysis and neurohypophysis, respectively (3). During this process, embryological cells of the adenohypophysis migrate through the craniopharyngeal canal to the nasopharynx or sphenoid sinus before uniting with the neurohypophysis in the sella turcica of the sphenoid bone. Infrasellar EPAs can thus be a result of persisting embryological cells along the migration pathway, manifesting in the sphenoid sinus or nasopharynx (1, 2). Alternatively, suprasellar EPAs may be derived from cells of the supradiaphragmatic portion of the pars tuberalis in the suprasellar region (1, 9, 13). Another potential explanation is aberrant embryological cells migrating through the craniopharyngeal canal to both extracranial (infrasellar—nasopharynx, sphenoid sinus, clivus) and intracranial (supra- and parasellar) areas (2, 13). The most common location for EPAs is the suprasellar and sphenoid sinus followed by the nasopharynx.

A literature review yielded 14 published case reports of ectopic TSHoma including our report (**Table 1**), using PubMed/Medline search terms “ectopic” AND “TSHoma/thyrotropinoma,” “ectopic” AND “TSH/thyrotropin” AND “pituitary,” “ectopic” AND “TSH/thyrotropin” AND “adenoma,” “ectopic” AND “TSH/thyrotropin” AND “pituitary” AND “adenoma.” The first case was reported in 1996; however, 50% of the cases were published in the past 5 years suggesting greater recent awareness of ectopic TSHoma. The majority of the cases were women (9/14) and the median age at diagnosis was 48 years (range 10–78 years). Unlike other EPAs, the nasopharynx was the most common location (9/14), followed by the suprasellar (3/14), sphenoid sinus (1/14), and sphenoid bone (1/14) which may represent a unique nasopharyngeal predisposition of ectopic TSHoma. Tumor size was recorded in 10 cases, with a median maximal diameter of 2.0 cm (range 0.4–3.0 cm, one microadenoma). Symptomatic thyrotoxicosis (11/14) was the most common complaint followed by diffuse goiter (9/14) reflecting TSH hypersecretion. Mass effects differed based on tumor location with nasopharyngeal/sphenoid sinus/sphenoid bone lesions presenting with nasal congestion (6/11) and suprasellar lesions with visual impairment (2/3). Biochemically, the vast majority of patients had elevated fT4 and fT3, while 50% had elevated TSH (100% of the cases had non-suppressed TSH). Primary thyrotoxicosis/Graves’ disease was the most common initial diagnosis (7/14) which led to inappropriate treatment with anti-thyroid drugs (8/14) and radioactive iodine ablation (2/14). Our patient is the first to undergo total thyroidectomy; however, this was performed for enlarging symptomatic goiter and suspicious nodule rather than inappropriate treatment of presumed Graves’ disease. The median diagnostic delay from the initial presentation was 2.5 years (range <1 to 18 years). The single most important investigation which led to the correct diagnosis was an MRI brain scan (11/14) with the majority ordered as part of the investigation algorithm for inappropriate TSH secretion to exclude TSHoma. Three cases, including ours, required the identification of the mass by otolaryngological examination for nasal congestion to facilitate the correct diagnosis. The ectopic TSHoma was initially missed on an MRI brain scan in one case; however, this was rectified within <1 year. Our patient, however, experienced a 7-year diagnostic delay despite the midline sphenoid bone mass being visible in the same sagittal and coronal slice as the pituitary gland under investigation, suggesting a lack of awareness for ectopic TSHoma. The interval growth of the ectopic TSHoma may reflect natural history or could have been exacerbated by thyroidectomy and the resulting impaired feedback inhibition by the thyroid hormone. All patients underwent tumor resection with positive biochemical and structural outcomes at a median follow-up of 9 months (range 2 months to 7 years), likely reflecting the lack of concern for postoperative hypopituitarism allowing for more extensive complete surgical resection. Histopathological confirmation of ectopic TSH-secreting neuroendocrine tumor was achieved in all cases supported by features of the neuroendocrine tumor and positive tumor cell immunohistochemistry for TSH (14/14).

**Table 1:** Summary of the 14 published cases to-date of ectopic TSHoma.

Author Year	Age Sex	Presentation	Initial investigations	Initial diagnosis	Initial treatment	Delay to diagnosis	LocationSize	Post-resection outcome and follow-up
Kumar et al. 2022	71 years Female	Intermittent dysphagia Diffuse goiter Re-presented with nasal congestion	Elevated fT4 High-normal fT3 High-normal TSH Normal-range TSH α-subunit Normal pituitary panel Negative *THRß* mutation	Inappropriate TSH secretion	Total thyroidectomy for progressive goiter/suspicious nodule	7 years	Sphenoid bone/clivus 3.0 cm × 2.3 cm	TFTs normalized at 5 months
Li et al. 2021	10 years Female	Sweats, heat intolerance Diffuse goiter	Elevated fT4 Elevated fT3 Normal-range TSH Normal pituitary panel Elevated SHBG Positive somatostatin suppression test Negative *THRß* mutation	Ectopic TSHoma	Surgical resection of the suprasellar mass	<1 year	Suprasellar 0.4 cm × 0.3 cm	Normalization of fT3, fT4, and TSH. Resolution of symptoms and no recurrence at 4 years
Ortiz et al. 2020	52 years Female	Weight loss, hyperdefecation Tachycardia, diffuse goiter	Elevated fT4 Elevated fT3 Elevated TSH Normal pituitary panel Elevated SHBG Positive somatostatin suppression test	Primary hypothyroidism	Thyroxine	2 years	Sphenoid sinus 2.4 cm × 2.4 cm	Initial recurrence requiring reoperation Subsequent normalization of TFTs and no structural recurrence at 2 years
Trummer et al. 2020	48 years Female	Palpitations, sweats	Elevated fT4 Elevated fT3 Elevated TSH Elevated alpha subunit/TSH ratio >1 Elevated SHBG Normal pituitary panel Positive TRH stimulation test	Inappropriate TSH secretion	Propylthiouracil Bisoprolol	1 year	Nasopharynx 2.0 cm × 1.8 cm	TFTs normalized No recurrence at 8 months
Kim et al. 2019	48 years Female	Palpitations, tremor, tachycardia Diffuse goiter	Elevated fT4 Elevated fT3 Normal-range TSH Elevated alpha subunit/TSH ratio >1 Diffusely increased uptake on thyroid uptake scan	Primary thyrotoxicosis	Methimazole Propylthiouracil Propranolol	4 years	Nasopharynx 1.1 cm × 0.8 cm	TFTs normalized No recurrence at 6 months
Hanaoka et al. 2018	41 years Male	Visual impairment	NR	Craniopharyngioma	Surgical resection of the suprasellar mass	<1 year	Suprasellar NR	Normal TFTs and no structural recurrence at 7 years
Yang et al. 2017	27 years Female	Palpitations Diffuse goiter Nasal congestion	Elevated fT4 Elevated fT3 Elevated TSH Normal pituitary panel Negative T3 suppression test	Primary thyrotoxicosis	Methimazole Metoprolol Radioactive iodine ablation	10 years	Nasopharynx NR	TFTs normalized No recurrence at 3-years.
Wang et al. 2016	46 years Male	Palpitations, sweats, weight loss Visual impairment Diffuse goiter	Elevated fT4 Elevated fT3 Elevated TSH	Primary thyrotoxicosis	Propylthiouracil	1 year	Suprasellar 1.5 cm × 1.2 cm	TFTs normalized No structural recurrence at 6 months
Song et al. 2014	41 years Male	Palpitations, weight loss, atrial fibrillation Diffuse goiter	Elevated fT4 Elevated fT3 Elevated TSH Negative TRH stimulation test Positive somatostatin suppression test	Primary thyrotoxicosis	Propylthiouracil Propranolol	<1 year	Nasopharynx 1.9 cm × 1.7 cm	TFTs normalized No structural recurrence at 4 years
Nishiike et al. 2014	46 years Male	Sweats, palpitations Headaches Diffuse goiter	Elevated fT4 Elevated fT3 Normal-range TSH Negative TRH stimulation test Diffusely increased uptake on thyroid uptake scan	Inappropriate TSH secretion	Surgical resection of the nasopharyngeal mass	3 years	Nasopharynx 1.4 cm	TSH normalized No structural recurrence at 3 years
Tong et al. 2013	49 years Female	Nasal congestion Symptoms of thyrotoxicosis	Elevated fT4 Elevated fT3 Elevated TSH Normal pituitary panel Positive octreotide suppression test	Sinusitis Grave’s disease	Methimazole	<1 year	Nasopharynx 2.0 cm × 2.0 cm	Recurrence-free at 3-month follow-up
Collie et al. 2005	50 years Female	Headaches, nasal congestion Non-suppressible TSH on thyroxine replacement	Non-suppressible TSH	Peripheral nerve sheath tumor	Surgical resection of the nasopharyngeal mass	NR	Nasopharynx 2.0 cm × 1.5 cm	TSH normalized No structural recurrence at 4 months
Pasquini et al. 2003	52 years Male	Sweats, palpitations, weight loss, atrial fibrillation Diffuse goiter Re-presented due to debilitating nasal congestion, rhinorrhea	Elevated fT4 Elevated fT3 Normal-range TSH Normal pituitary panel Diffusely increased uptake on thyroid uptake scan	Grave’s disease	Methimazole	18 years	Nasopharynx NR	TFTs normalized initially Nasal congestion resolved Rising fT3 and fT4 at 10-month follow-up suspicious for incomplete resection
Cooper et al. 1996	74 years Female	Anxiety, tremors, weight loss Re-presented with nasal congestion	High-normal fT4 High-normal fT3 High-normal TSH Elevated alpha subunit/TSH ratio >1 Normal-range SHBG Positive TRH stimulation test	Grave’s disease	Propylthiouracil Radioactive iodine ablation	9 years	Nasopharynx NR	TFTs normalized Nasal congestion resolved Recurrence-free at 2-month follow-up

fT4, free thyroxine; fT3, free tri-iodothyronine; TSH, thyroid-stimulating hormone; THR, thyroid hormone receptor; TRH, thyrotropin-releasing hormone; SHBG, sex hormone-binding globulin; NR, not recorded; TFT, thyroid function tests.

The differential diagnosis for inappropriate TSH secretion includes assay interference, thyroid hormone resistance, familial dysalbuminemic hyperthyroxinemia (FDH), and TSHoma (18). Laboratory assay interferences should first be excluded, such as the presence of heterophile antibodies, thyroid hormone antibodies, and high biotin levels (19). A useful approach to exclude assay interference is to repeat TFTs using a different assay in a different laboratory and, if other common differentials are excluded, to consider consultation with the biochemical department for exclusion of interfering antibodies. FDH occurs *via* autosomal dominant inheritance of pathogenic variants in the gene encoding albumin. The resulting mutant albumin has a higher binding affinity to T4 resulting in two- to three-fold elevated total T4 and mildly elevated total T3 concentrations. The mutant albumin also has greater binding to labeled T4 analogs, the assay tracer, and causes more antibodies to bind fT4 instead, giving abnormally high fT4 measurements (20). Thyroid hormone resistance is caused by autosomal dominant inheritance of mutations in the thyroid hormone receptor (THR) genes *THRα* and *THRß* which encode intranuclear T3 receptors, resulting in T3 receptor dysfunction. *THRß* mutation is by far the most prevalent subtype with biochemical findings of elevated fT4 and inappropriately normal or elevated TSH levels, biochemically indistinct from TSHoma (21). To help differentiate between the two conditions, family history should be sought and germline DNA sequencing for *THRß* mutations performed. Investigations that support the diagnosis of TSHoma over thyroid hormone resistance include elevated α-subunit/TSH ratio (>1.0) and serum sex hormone-binding globulin level (peripheral tissue marker of thyroid hormone action). Dynamic investigation results include the positive somatostatin suppression test (due to predominantly somatostatin receptor 2 expression), negative TRH stimulation test, and negative T3 suppression test (21, 22). It is unclear due to the rarity of cases whether the same conclusions can be drawn regarding ectopic TSHoma. However, we have observed certain clinical, biochemical, and radiological characteristics from the published case reports in comparison to pituitary TSHoma (**Table 2**).

**Table 2:** Clinical, biochemical, and radiological characteristics of pituitary and ectopic TSHoma.

	Pituitary TSHoma	Ectopic TSHoma
**Clinical presentation**	Hyperthyroidism, goiter Hypopituitarism	Hyperthyroidism, goiter ENT/visual symptoms
**Location**	Pituitary	Nasopharynx/suprasellar/sphenoid
**Size**	Macro > micro	Macro > micro
**Thyroid function**	High FT4, high FT3, normal/high TSH	High FT4, high FT3, normal/high TSH
**Alpha subunit/TSH molar ratio**	Increased/normal	Increased/normal
**Sex hormone-binding globulin (SHBG)**	Increased/normal	Increased/normal
**Somatostatin suppression test**	Positive	Positive
**MRI pituitary**	Pituitary adenoma	Normal pituitary
**Surgical cure**	<60% cure rate with macroadenoma	93% cure rate

TSH, thyroid-stimulating hormone; MRI, magnetic resonance imaging; fT4, free thyroxine; fT3, free tri-iodothyronine; ENT, ear nose & throat.

Previous cases of ectopic TSHomas have mostly been reported as a nasopharyngeal mass, and this is the first case where it was discovered in the sphenoid bone/clivus. Primary clival EPAs, as seen in our patient, are defined as EPAs being contained within the clivus and have been reported to be exceedingly rare (2). A clival tumor, however, has a broad differential diagnosis, including chordoma, chondrosarcoma, intraosseous meningioma, craniopharyngioma, lymphoma, myeloma, and solid organ metastases, with chordoma being the most prevalent (1). Clival EPAs have also been observed to be associated with malignant transformation, bone invasion, and a concurrent empty sella (1, 4), of which only bone invasion was observed in our patient. In addition, our patient also has a concurrent history of PTC, and this has only been described in another case report of ectopic TSHoma. Given the presence of goiter in other patients with ectopic TSHoma, the ectopic TSH is promoting the growth and proliferation of thyroid follicular cells, which may explain the association with PTC in our case and another case reported by Yang et al. (12). However, our patient’s underlying Hashimoto’s thyroiditis may also have been a contributing factor to PTC pathogenesis. Furthermore, incidental micro-PTC in thyroidectomy specimens is not uncommon, and the clinical relevance of this finding is unclear (23–25). To our knowledge, there is insufficient evidence at this stage to directly link ectopic TSHoma with PTC.

In summary, we present the first reported case of sphenoid bone ectopic TSHoma. Initial investigations revealed elevated fT3 and fT4, with an inappropriate high-normal TSH indicative of inappropriate TSH secretion. The initial diagnosis was not obtained in the setting of negative germline DNA testing for *TRHß* gene mutation, normal post-menopausal range TSH α-subunit level, and a pituitary MRI scan that was reported as “normal.” The patient underwent total thyroidectomy 1 year later for progressive symptomatic goiter and suspicious nodule which revealed multifocal micro-PTC. Six years later, she presented with nasal congestion which directed the investigation to her nasopharynx, facilitating the discovery of a sphenoid bone mass. After surgical resection and histopathological tumor examination, the final correct diagnosis of ectopic TSHoma was obtained. Retrospective analysis of the initial pituitary MRI scan 7 years prior revealed that the sphenoid bone mass had been present since her initial presentation. Although ectopic TSHoma is exceedingly rare, we advocate that this diagnosis should be considered in patients with inappropriate TSH secretion and exclusion of other more common differential diagnoses such as assay interference, thyroid hormone resistance, and pituitary TSHoma, to minimize the diagnostic and therapeutic delay. Ectopic TSHoma may be clinically and biochemically indistinguishable from pituitary TSHoma, and one should have high clinical suspicion if no pituitary adenoma is identified on imaging and to look outside the sella turcica for an ectopic tumor in the nasopharynx, suprasellar, sphenoid sinus, and sphenoid bone.

## Patient perspective

The patient declined to provide her perspective on the case report; however, she provided signed written informed consent for this report to be published.

## Data availability statement

The original contributions presented in the study are included in the article/supplementary material. Further inquiries can be directed to the corresponding author.

## Ethics statement

Ethical review and approval was not required for the study on human participants in accordance with the local legislation and institutional requirements. The patients/participants provided their written informed consent to participate in this study. Written informed consent was obtained from the individual(s) for the publication of any potentially identifiable images or data included in this article.

## Author contributions

SK conceptualized, drafted, and critically reviewed the manuscript. CP and HN assisted in drafting the manuscript. TD managed the patient and critically reviewed the manuscript. All authors contributed to the article and approved the submitted version.

## Acknowledgments

The authors acknowledge Dr. Peter Earls, Anatomical Pathology Department, SydPath (St Vincents Pathology), NSW, Australia, for his assistance in the preparation and interpretation of tumor histopathology slides.

## Conflict of interest

The authors declare that the research was conducted in the absence of any commercial or financial relationships that could be construed as a potential conflict of interest.

## Publisher’s note

All claims expressed in this article are solely those of the authors and do not necessarily represent those of their affiliated organizations, or those of the publisher, the editors and the reviewers. Any product that may be evaluated in this article, or claim that may be made by its manufacturer, is not guaranteed or endorsed by the publisher.
